# Evaluating packaged ice safety using culture-based and metagenomic methods

**DOI:** 10.1099/mic.0.001780

**Published:** 2026-10-08

**Authors:** Sarah Cromb, Daniel Mayboca-Padilla, Ruhani Bedi, Claudia Narvaez-Bravo

**Affiliations:** 1 Faculty of Agricultural and Food Sciences, University of Manitoba, Winnipeg, MB R3T 2N2, Canada

**Keywords:** packaged ice, indicator micro-organisms, *Pseudomonas lactis*, foodborne pathogens, metagenomics, biofilms, food safety

## Abstract

Ice can serve as a vector for microbial contamination, presenting potential risks to public health. This study aimed to evaluate the microbial quality and safety of commercially available ice in Manitoba as well as to characterize the microbiome of ice cubes and assess the presence of antimicrobial resistance (AMR) genes. Samples from five different brands, four non-International Packaged Ice Association (IPIA)-accredited and one IPIA-accredited brand, were collected from retail stores between May and August 2024. Ice samples were tested using membrane filtration for total plate count, *Escherichia coli*, coliforms, *Pseudomonas*, yeasts, moulds and *Enterococcus* and screened for *Listeria monocytogenes* and *Salmonella*. Separate filtration samples were collected for metagenomic analysis. Membrane filtration results showed that 12.8% of samples from non-IPIA-accredited brands failed to meet IPIA standards for total coliforms, whereas 100% of samples from the IPIA-accredited brand complied. All samples tested negative for foodborne pathogens when conventional culture-based methods were used. However, metagenomic sequencing revealed the presence of pathogen-associated DNA. A diverse resistome was identified across all brands, including the IPIA-accredited sample, with genes conferring resistance to *β*-lactams, aminoglycosides and biocides. Recovered *Pseudomonas* isolates were identified as *Pseudomonas lactis*, a psychrotrophic species capable of growth at refrigeration temperatures. Biofilm assays demonstrated that recovered *P. lactis* formed biofilms at both 4 and 10 °C, with significantly greater biofilm formation observed at 4 °C. Our findings demonstrate consistent compliance among the IPIA-accredited brand, while variability and failures occurred among non-accredited producers. The detection of cold-adapted, biofilm-forming bacteria such as *Pseudomonas* spp., which can reduce sanitizer efficacy and protect co-existing micro-organisms, including potential pathogens harbouring AMR genes, highlights the need for targeted environmental monitoring using key indicator organisms such as *Pseudomonas* spp. Broader adoption of IPIA standards could strengthen microbial control practices and enhance consumer protection within the Canadian packaged ice industry.

## Data availability

The metagenomic reads from the ice samples analyzed in this study were deposited in the NCBI Sequence Read Archive (SRA) under BioProject ID PRJNA1328825. The *Pseudomonas* sequencing data were deposited under BioProject ID PRJNA1294336. The full list of accession numbers for the datasets used in this study is provided in Table S1.

## Introduction

In Canada, contaminated food and beverages cause an estimated 4 million cases of foodborne illness, 11,600 hospitalizations and 238 deaths annually [1]. A substantial proportion of these cases remain unattributed to a definitive source. Although ice is a widely consumed food product, it is rarely prioritized in epidemiological tracebacks due to a false perception of safety associated with its frozen state [2]. Water and ice can be a source of microbial contamination, and several outbreaks of foodborne illnesses have been linked to the consumption and usage of contaminated ice cubes [3, 4]. Recognizing the risk, the World Health Organization (WHO) recommends that ice intended for human consumption or food contact should adhere to the same quality and safety standards as drinking water [5]. Contamination of drinking water can occur through the introduction of human and animal waste from runoff, inadequate water treatment or poor adherence to standardized cleaning and sanitation protocols [6, 7]. Furthermore, edible ice can pose a direct transmission risk, serving as a vehicle for the introduction of micro-organisms through poor handling, unsanitary equipment or direct contact with source water, as recent metagenomic monitoring has shown that source water naturally carries significant bacterial populations and resistance genes [8]. Indicator micro-organisms, such as coliforms, *Escherichia coli* and Enterococci, are used to assess the microbiological quality of ice, as their presence can indicate sources of contamination or inadequate sanitation during production or handling [9]. Micro-organisms commonly associated with water contamination, including *Cryptosporidium*, *E. coli* and *Pseudomonas*, are also frequently screened in ice, along with indicator organisms such as coliforms, *Enterococci*, yeasts and moulds [4–6, 10]. Beyond indicator organisms, foodborne pathogens are also considered an important component of the microbiological safety of packaged ice. Consequently, studies evaluating the microbiological quality of edible ice routinely assess pathogens such as *Salmonella* spp. and *Listeria monocytogenes*, as their presence is not permitted under microbiological standards [11]. Although these pathogens are infrequently detected, *Salmonella* has been reported in retail ice samples, highlighting the importance of maintaining hygienic production and handling practices [5].

In addition to source water contamination, persistent microbial contamination in ice production systems could also arise from biofilm formation on food-contact and plumbing surfaces. Biofilms are structured microbial communities embedded within a self-produced extracellular matrix that enables micro-organisms to adhere to surfaces and persist under adverse conditions, including low temperatures and routine sanitation practices [12]. Psychrotrophic bacteria, such as members of the *Pseudomonas* genus, are particularly well adapted to biofilm formation under cold, low-nutrient conditions typical of ice manufacturing and storage environments [13]. Consequently, biofilm development may be an underlying mechanism contributing to recurring or persistent microbial detection in ice products, even when source water meets quality standards. Currently, there is a significant knowledge gap regarding the presence and persistence of biofilm-forming micro-organisms in edible ice manufacturing environments.

In addition to microbial persistence, metagenomic analysis studies have shown that the environmental resistome in food production systems may act as reservoirs and transmission routes for antimicrobial resistance (AMR) genes, facilitating their transfer to minimally processed ready-to-eat foods [14–16]. Furthermore, while freezing conditions can limit microbial growth, they do not eliminate bacteria or their genetic material, thereby allowing genetic elements, including AMR genes, to persist [17, 18]. As a result, metagenomic analyses of ice microbiomes have identified a diverse reservoir of AMR genes (often localized on mobile genetic elements), which confer resistance to commonly used antibiotics, including *β*-lactams, tetracyclines and sulphonamides [19–21]. Ultimately, the presence of AMR genes in edible ice highlights the importance of maintaining high water quality standards, hygienic processing conditions, effective equipment sanitation and comprehensive environmental monitoring programmes. It also highlights the need for enhanced surveillance strategies to better understand the potential food safety and public health implications associated with AMR in edible ice production environments. Given its widespread use in the food service industry, ice is commonly used as a cooling agent for beverages, particularly during the summer months, as well as in food preparation and as an ingredient in various products [22] In the USA, 80% of packaged ice is purchased between May and September, with the average American buying four bags annually, according to estimates from the US Food and Drug Administration (FDA) [23]. In Canada, the Canadian Food Inspection Agency (CFIA) states that ice used in food preparation (as a cooling agent or an ingredient) should be regulated as a food. Similarly, the FDA mandates that packaged ice labels disclose the water source. Yet, despite these requirements, manufacturing facilities are not consistently inspected due to budget constraints [23].

While both the CFIA and the US FDA provide guidance for ice manufacturers, primarily through Good Manufacturing Practices (GMPs) and sanitation-related Standard Operating Procedures (SOPs), the regulatory oversight of ice varies by jurisdiction and intended use. In Canada, ice sold and distributed solely within a province is generally regulated at the provincial or municipal level, rather than federally, unless it is produced for interprovincial or international trade or for use in federally registered establishments. As a result, the ice samples analysed in this study, which were sold exclusively within the province, were not subject to direct federal CFIA regulatory oversight. However, businesses committed to producing safer products and maintaining a strong food safety culture often voluntarily pursue third-party accreditation through industry organizations. For edible ice, in both the USA and Canada, this is commonly achieved through the International Packaged Ice Association (IPIA). In Canada, ice producers that are members of the Canadian Association of Ice Industries (CAII) are required to comply with IPIA guidelines and to undergo food safety audits in accordance with the Packaged Ice Quality Control Standards (PIQCS) [24]. The IPIA strives to educate retailers, regulators and consumers on food safety and promotes the use of PIQCS, which includes Hazard Analysis and Critical Control Point (HACCP) programmes, to accredit ice brands. PIQCS establishes microbiological criteria for packaged ice, including an aerobic plate count of ≤500 c.f.u. ml^−1^, the absence of total coliforms (including *E. coli*) and zero tolerance for *Salmonella* spp. and *L. monocytogenes* [23]. In Canada, limited research has examined the microbial quality and safety of commercially available ice, particularly regarding the impact of industry accreditation on microbial standards and food safety outcomes, which is critical information needed for accurate risk assessment and the development of effective regulatory and preventive strategies. This study aimed to (a) assess the microbial quality and safety of commercially available ice in Manitoba, (b) compare accredited and non-accredited ice cube producers and (c) characterize the ice microbiome, with particular emphasis on foodborne pathogens and AMR genes. Additionally, (d) *Pseudomonas* spp. strains recovered during sample analysis were further characterized to determine their AMR profiles and biofilm-forming capacity.

## Methods

### Sampling

Samples included five packaged ice brands available in Manitoba, Canada, purchased from 26 retail locations across the province. These included one IPIA-accredited brand and four non-accredited brands. For anonymity purposes, the four non-IPIA brands were labelled Brand A–D and the IPIA-accredited brand as Brand E. Between May and August, a total of 178 bags were collected, comprising 133 bags from non-accredited brands and 45 bags from the IPIA-accredited brand. At each sampling occasion, two to three bags of ice from different brands, if available, were randomly selected from different positions within the freezer and were processed independently as individual samples. During sample collection, ice bags were kept separate from each other in individual plastic bags and placed in a cooler. In the laboratory, the exterior of each ice bag was sanitized using 70% isopropyl alcohol before opening. Ice samples were aseptically transferred into Whirl-Pak^®^ bags using sterile scissors and tongs. Ice samples were allowed to melt at 4–5 °C for 2–4 days and were processed immediately after melting. Refrigerated thawing was used to minimize changes in the microbial population during sample preparation and to limit microbial growth before analysis [10]. Subsequently, each sample was tested for its total and free chlorine content using the Hach DR300 Pocket Colorimeter chlorine kit (Hach, London, ON, Canada) following the manufacturer’s instructions.

### Membrane filtration for microbial quality and indicator micro-organisms

After melting, 100 ml of the sample was filtered using the Neogen filtration system (Neogen Corporation, Lansing, MI, USA) [8]. The samples were analysed for total aerobic bacteria (total plate count), yeasts, moulds, coliforms, Enterococci and *Pseudomonas* spp. For all indicator organisms except Enterococci, individual filters were placed in Neogen ampouled media containing 2 ml of selective broth to support the growth of the target micro-organisms. Specifically, m-TGE broth was used for total plate count, m-ENDO broth for coliform enumeration and MI broth for the detection of both coliforms and *E. coli*. All three were incubated at 35 °C for 18–24 h. *Pseudomonas* were enumerated using *Pseudomonas* broth and were incubated at 30 °C for 48 h. The m-Green broth was used for yeasts and moulds and was incubated at 25–27 °C for 72 h. After incubation, all plates were enumerated and results were recorded. *Pseudomonas* colonies recovered from the IPIA-accredited brand were isolated and frozen in 15% glycerol at –80 °C for further analysis, including biofilm formation assays.

### Enterococci

To enumerate Enterococci, 10 ml of 0.1% buffered peptone water (BPW; BD Difco, Franklin Lakes, NJ, USA), was added to the Neogen filter after filtration, then sonicated for 1 min. The sample was mixed five times clockwise and counterclockwise following sonication to ensure homogenization. Then, 0.1 ml of the BPW from the filter was spread plated onto four Bile Esculin agar plates (Hardy Diagnostics, Criterion, Santa Maria, CA, USA) using a plastic L-shaped cell spreader (Avantor, VWR, Radnor, PA, USA). The plates were incubated for 24–48 h at 35 °C.

### Pathogen detection

*Salmonella* detection was performed according to the FDA Bacteriological Analytical Manual (BAM) [25] and *L. monocytogenes* following the ISO 11290-1 [26] with some modifications. The filter for the detection of *L. monocytogenes* and *Salmonella* was enriched with universal broth (Oxoid, Thermo Fisher Scientific, Nepean, ON, Canada) and incubated at 35 °C for 24 h. This step was included due to the low expected microbial load of packaged ice and the potential for cold-induced cellular injury, and samples were initially enriched in to facilitate recovery of low numbers of potentially stressed or injured bacterial cells. Following enrichment, 1 ml of broth was transferred to Rappaport-Vassiliadis (RV) broth (HiMedia, Kelton, PA) and tetrathionate (TT) broth (BD Difco, Franklin Lakes, NJ), for *Salmonella*. For *Listeria*, 1 ml of broth was transferred to Half-Fraser broth (Hardy Diagnostics, Criterion, Santa Maria, CA). RV and TT were incubated at 35 °C for 24 h, and Half-Fraser was incubated at 30 °C for 48 h. After incubation, a loopful from RV and TT was streaked onto Xylose Lysine Tergitol 4 (XLT4; Hardy Diagnostics, Santa Maria, CA), supplemented with XLT4 agar supplement (Difco, Sparks, MD) and Hektoen Enteric (HE; BD Difco, Franklin Lakes, NJ). For *L. monocytogenes*, a loopful Half-Fraser was streaked onto Oxford Listeria agar (Oxoid, Thermo Fisher Scientific, Nepean, ON). Following this non-selective enrichment, 1 ml of broth was transferred to Half-Fraser broth (Hardy Diagnostics, Criterion, Santa Maria, CA) for selective enrichment of *Listeria* and incubated at 30 °C for 48 h. A loopful of the Half-Fraser enrichment was subsequently streaked onto Oxford *Listeria* agar (Oxoid, Thermo Fisher Scientific, Nepean, ON) and incubated at 35 °C for 24 h. Presumptive colonies were confirmed using the 3M Molecular Detection Assay – *L. monocytogenes* (3M Food Safety, now Neogen Corporation, Lansing, MI, USA), according to the manufacturer’s instructions. All agar plates were incubated at 35 °C for 24 h. Presumptive colonies were subsequently confirmed using the Neogen Molecular Detection Assay – *Salmonella* and the 3M Molecular Detection Assay – *L. monocytogenes* (3M Food Safety, now Neogen Corporation, Lansing, MI, USA), according to the manufacturer’s instructions.

### Genomic preparation and sequencing for microbiome and *Pseudomonas* isolates

Ice samples were subjected to metagenomic characterization of the enriched culturable bacterial community associated with each brand. Therefore, to obtain sufficient microbial biomass for DNA extraction, ice samples from each brand were combined to obtain one 500 ml composite sample. Each composite was filtered through a Neogen filter, supplemented with an m-TGE ampoule and incubated for 24 h at 35 °C. Consequently, each brand was represented by a single composite sample for metagenomic analysis, with no biological replication at the metagenomic level. Methods for bacterial fraction recovery and DNA extraction were adapted from Jankowski *et al*. [27], with minor modifications. Briefly, the filters were removed using a sterile scalpel and placed into Falcon tubes containing 15 ml of 1× PBS-Tween20 (Thermo Scientific, Rockford, IL) solution and homogenized at 2500 rpm for 15 min. The filters were removed and centrifuged for 15 min at 3,300 ***g***. The pellet was resuspended and transferred to a Disrupter Tube for DNA extraction using the EZNA Soil DNA kit (Omega Bio-tek, Norcross, GA) as per the manufacturer’s instructions.

*Pseudomonas* spp. consistently isolated from Brand E were preserved in 15% glycerol and stored at −80 °C for subsequent sequencing and genomic analysis. Frozen isolates were grown at 25 °C for 48 h in trypticase soy broth (BD Difco, Franklin Lakes, NJ, USA). Genomic DNA was extracted using the Gram-negative protocol from the DNeasy Blood and Tissue Kit (Qiagen, Hilden, Germany) and followed as per the manufacturer’s instructions. DNA concentrations were determined using the Qubit High Sensitivity assay kit (Thermo Fisher Scientific, MA, USA).

DNA libraries were prepared using the sparQ DNA Frag and Library Prep Kit (Quantabio, Beverly, MA), following the manufacturer’s instructions. The prepared DNA libraries were sent to Genome Quebec Innovation Centre (Montreal, Canada) for sequencing using the Illumina NovaSeq X Plus platform. Following sequencing, reads were quality trimmed using fastp (v0.24.0) with default parameters to remove adapter sequences as well as polgG sequences that occur at the end of some reads [28].

### Bioinformatics of metagenomic reads

The raw, trimmed metagenomic reads were uploaded to Galaxy Europe platform [29]. Kraken2 (v2.1.3+galaxy1) assigned taxonomic labels with default parameters using the Prebuilt Refseq indexes: PlusPF-16 database (v2024-01-12). Bracken (v3.1+galaxy0) was used to re-estimate the abundance of bacteria at the genus level from the Kraken2 report, using the PlusPF-16 (v2024-01-12, K-mer:35, read: 100) database. The microbial diversity was plotted using RStudio v2024.12.1.563 [30] using the *ggplot2* package v3.5.1 [31]. Metagenomic reads were assembled into contigs using the default parameters of metaSPAdes (v4.2.0), and the resulting contigs fasta files were used for downstream analysis. AMR genes were identified within the assembled metagenomes using the National Center for Biotechnology Information (NCBI) AMRFinderPlus tool (v3.12) with a minimum identity threshold of 90% and coverage of 60% [32]. Additionally, biocide and metal resistance genes were identified within the metagenomic assemblies by comparing the assembled contigs against the experimentally confirmed Antibacterial Biocide and Metal Resistance Genes Database BacMet (v2.0) [33] using DIAMOND blastp (v2.1.13) [34] in very sensitive mode. For these metagenomic samples, an e-value of 1e-6 and a minimum identity and coverage of 60% were applied as recommended for water metagenomic samples [35].

A network analysis was conducted to visualize the biocide and AMR genes across the sampled ice brands using Python (v3.14.4) with the *network* package in the *pandas* library [36]. The metagenomic reads were deposited into the Sequence Read Archive (SRA) of NCBI under the BioProject ID PRJNA1328825.

### Bioinformatics of isolated *Pseudomonas*

Raw reads from *Pseudomonas* isolates were assembled using unicycler v0.4.8 [37]. Assemblies were annotated using RASTtk [38] via the BV-BRC platform [39]. The average nt identity (ANI) was calculated to assess genomic relatedness among *Pseudomonas lactis* isolates. Pairwise ANI values were generated using FastANI (v1.34) [40] with default parameters. The resulting ANI output file was processed in RStudio (v4.4.2) [30] to construct a symmetrical similarity matrix. A heat map was generated using the *pheatmap* package (v1.0.13) [41], where each cell represents the pairwise ANI value between isolates. A blue gradient colour palette (99.93–100%) was applied to visualize subtle differences in nt identity among isolates. Additionally, a core SNP analysis was conducted in the Galaxy platform where whole genome sequencing (WGS) reads for each isolate were mapped against the reference genome *P. lactis* DSM 29167. Initial read alignment, variant identification and the filtering of low-quality calls were performed using Snippy v4.6.0 [42]. Following the individual variant calling, the outputs for all isolates were aggregated using Snippy-core v4.6.0 [43] (reference). Finally, to quantify the pairwise SNP distances among the isolates, the core SNP alignment was processed using snp-dists v0.8.2 [44].

The detection of biocides, metal resistance and biofilm genes was performed for the isolated *Pseudomonas* strains, using a read-based bioinformatic workflow on the Galaxy Europe platform [29]. CDSs were predicted from the assembled Illumina genomes using Prodigal (v2.6.3) [45]. The resulting protein sequences were compared against the Bacteria Biofilm Sequence Database (BBSdb) [46] and the experimentally confirmed BacMet database (v2.0) [33], using DIAMOND blastp (v2.1.13) [34] in sensitive alignment mode. The search parameters included the following: e-value of 1e-6, a minimum sequence identity of 90%, a minimum query coverage of 80% and a minimum subject coverage of 80%.

The presence of AMR genes in the *Pseudomonas* isolates was determined using the abriTAMR [47] workflow (v1.0.19+galaxy1) available on the Galaxy platform [29]. This workflow incorporates the AMRFinderPlus tool developed by the NCBI to identify acquired resistance genes, point mutations and stress response determinants in bacterial genomes. Assembled genome files were provided as input, and default parameters were used for gene prediction and annotation.

The pan-genome construction and gene presence–absence analysis of the *Pseudomonas* isolates was performed using Roary (v3.1.0+galaxy3) [48] on the Galaxy platform. Annotated genome files (GFF3 format), previously generated through Prokka (v1.14.6+galaxy1) [49], were used as input. Roary was executed with a minimum blastp percentage identity of 95% to cluster orthologous genes. Core genes were defined as those present in 99% of the isolates, while genes present at lower frequencies were assigned to the accessory genome. The phylogenetic tree generated by Roary was then used to visualize the distribution of the identified genes from BacMet using the Interactive Tree of Life (iTOL) tool [50]. The reads were deposited into the SRA of NCBI BioProject ID PRJNA1294336 for the *Pseudomonas* strains.

### *Pseudomonas* biofilm formation assay

Interestingly, *Pseudomonas* strains were consistently recovered from ice samples obtained from the IPIA-accredited Brand E; these isolates were selected for further characterization. *Pseudomonas* spp. isolates were subjected to biofilm formation assays, using the crystal violet (CV) method, under low-temperature conditions (4 and 10 °C) to simulate refrigerated and cold storage environments. Frozen stock cultures were grown in Luria–Bertani nutrient broth without salt (LBNS; tryptone 10 g l^−1^ and yeast extract 5 g l^−1^) and incubated at room temperature for 48 h. Cultures were adjusted to 0.5 McFarland standard and further diluted in LBNS to obtain a final concentration of 10⁶ c.f.u. ml^−1^. A 20 µl aliquot of the culture was transferred into microplate wells containing 180 µl of LBNS. Plates were incubated at 4 and 10 °C for 8 days to allow biofilm development, representing refrigeration and production temperatures [51]. *Raoutella terrigena* was included as a control strain due to its documented strong biofilm formation at 10 °C [52]. Following incubation, the supernatant was aspirated, and the wells were washed three times with 300 µl of 1.25% Butterfield’s Phosphate Buffer (BPB; Hardy Diagnostics Inc., Santa Maria, CA, USA) using a microplate washer (405 LS, BioTek, Winooski, VT, USA). Plates were air-dried for 10 min; then, 200 µl of absolute methanol was added to each well and left to stand for 15 min to fix the attached biofilm. After fixation, methanol was removed, and plates were air-dried for an additional 15 min. Biofilms were stained with 200 µl of 0.1% CV (Sigma-Aldrich, St. Louis, MO, USA) for 20 min, then washed three times with 200 µl BPB and air-dried for 15 min. Finally, 200 µl of 85% ethanol was added to dissolve the residual CV, then the plate was shaken for 10 s [53] and the OD was measured at 630 nm using a microplate reader (ELx800, BioTek Instruments Inc., Winooski, VT, USA). The biofilm classification cutoff was performed as previously described [53]. Briefly, biofilm classification thresholds were determined using the negative control, where the OD cut-off (ODc) was defined as the mean OD of the negative control plus three sd. Biofilm-forming capacity was subsequently categorized based on measured OD values as follows: OD ≤ODc indicated a non-biofilm former; ODc <OD≤2 ODc indicated a weak biofilm former; 2ODc <OD≤4 ODc indicated an intermediate biofilm former; and OD >4 ODc indicated a strong biofilm former. A one-way ANOVA was conducted in RStudio (v2024.12.1.563 [30) to assess significant differences between samples incubated at 4 and 10 °C.

## Results and discussion

### Microbial indicators and compliance with IPIA standards

Table 1 summarizes the microbial compliance of IPIA- and non-IPIA-accredited ice samples relative to IPIA standards. All samples from Brand E, the IPIA-accredited brand, met the PIQCS set by the IPIA. Similarly, Brand B, a non-IPIA-accredited brand, met these standards as well. However, Brands A, C and D, also non-IPIA-accredited, failed to meet the IPIA’s total coliform standards. Specifically, Brand B exceeded the acceptable limit of 500 c.f.u. ml^−1^ in one sample; 4.3% of Brand A’s samples and 37.5% of Brand C’s samples tested positive for coliforms, exceeding the acceptable limit of 0 c.f.u. ml^−1^ [11]. The detection of coliform bacteria in these brands suggests contamination from environmental sources such as soil, plants or faecal matter of animal or human origin and may also indicate inadequate hygiene practices during production or handling [54, 55]. In comparison, all the samples from Brands B, D and E met the standards for coliforms set by the IPIA, suggesting better sanitation practices. Presumptive colonies found from the IPIA and non-IPIA brands tested negative for *Salmonella* and *L. monocytogenes*.

**Table 1.** Comparison of microbiological compliance of IPIA- and non-IPIA-accredited packaged ice samples based on the US PIQCS set by the IPIA

			Non-complying sample (%)			
Packaged ice	Brand	No. of samples	TPC*	Total coliform^†^	Pathogen^‡^	Total
Non-IPIA accredited	A	46	2.2 (1)	4.3 (2)	0	6.5 (3)
	B	27	0	0	0	0
	C	40	17.5 (7)	37.5 (15)	0	55 (22)
	D	20	5 (1)	0	0	5 (1)
IPIA accredited	E	45	0	0	0	0

*Unacceptable if greater than 500 c.f.u. ml−1.

†Total coliforms including *E. coli*; acceptable limits are 0 c.f.u. ml−1.

‡*L. monocytogenes* and *Salmonella* were the pathogens tested: zero tolerance for the presence of pathogens [11].

TPC, Total Plate Count.

The average free chlorine concentration measured in both IPIA and non-IPIA ice products was ~0.02 mg l^−1^, with a maximum concentration of ~0.03 mg l^−1^. In comparison, drinking water regulations recommend maintaining a free residual chlorine concentration of ~0.2 mg l^−1^ within distribution systems to ensure microbial control, with a maximum allowable residual of 4 mg l^−1^ [56].

### Yeast and moulds

Although yeasts and moulds are not included in the microbiological criteria outlined by the IPIA, they were evaluated in this study to provide a broader assessment of microbial quality in pre-packaged ice. As shown in Table 2, mean mould counts for Brands A–E were 5.35, 3.74, 17.6, 4.6 and 0.578 c.f.u./100 ml, respectively, while mean yeast counts were 6.74, 13.9, 6.58, 125 and 2.29 c.f.u./100 ml, respectively. Overall, most brands exhibited relatively low average fungal counts; however, substantial variability was observed among brands, particularly for yeast counts in Brand D. This brand showed an elevated mean yeast concentration and the largest sd, reflecting highly heterogeneous contamination levels across samples, with a maximum yeast count of 1,190 c.f.u./100 ml. It is noteworthy that, while yeasts and moulds are not monitored under IPIA guidelines, the IPIA-accredited brand (Brand E) exhibited the lowest mean counts for both organisms. Although causality cannot be inferred, this observation suggests that adherence to IPIA accreditation standards, including monitored sanitation and cleaning practices, along with other food safety programmes such as HACCP, likely contributed to improved overall microbial control, including for non-regulated organisms such as yeasts and moulds.

**Table 2.** Mean yeast and mould counts (c.f.u./100 ml) in IPIA- and non-IPIA-accredited ice brandsValues are presented as mean±sd.

Packaged ice	Brand	Yeast	Mould
Non-IPIA accredited	A	6.74±11.40	5.35±7.11
	B	13.93±15.64	3.74±2.88
	C	6.58±18.95	17.55±50.66
	D	124.90±294.25	4.60±14.07
IPIA accredited	E	2.29±10.50	0.58±1.36

These findings are consistent with previous reports documenting substantial variability in fungal contamination of ice and water-derived products. Francesca *et al*. [57], reported wide ranges in yeast and mould levels among ice samples collected from home level, bars and pubs and industrial manufacturing plants within the Palermo province (Sicily, Italy). Fewer ice samples were positive for yeasts; however, with the exception of one sample, yeast populations exceeded mould populations whenever yeasts were detected. Yeast counts ranged from 0 to 383.8±20.5 c.f.u./100 ml, whereas mould counts ranged from 0 to 195.3±21.4 c.f.u./100 ml [42]. These findings are consistent with our observations, where yeast counts frequently exceeded mould counts and substantial variability was observed between samples. Our findings suggest that yeasts and moulds may serve as useful microbial indicators for monitoring the hygienic quality of edible ice and the effectiveness of sanitation practices during production and handling. Based on conventional culture-based methods incorporating enrichment and selective media, *L. monocytogenes* and *Salmonella* were not detected in any of the samples.

### Metagenomic detection in relation to regulatory testing

Metagenomic sequencing enables the detection of DNA in a sample, including bacteria, viruses, fungi and protozoa, without the need for culturing. This approach provides a more comprehensive assessment of the microbial community present, as it can detect organisms that are present in low numbers or in a viable but non-culturable (VBNC) state, which can be missed by traditional culture-based methods. However, in this study, because insufficient DNA was recovered from the individual ice samples, the metagenomic analysis was performed on the enriched bacterial communities. Therefore, the sequencing results presented in Table 3 represent the enriched bacterial community, with microbial diversity expressed as a percentage of total reads. Sequencing revealed the presence of *E. coli*, coliforms and *Pseudomonas aeruginosa* DNA in all five brands. Additionally, DNA sequences assigned to *Salmonella* were detected in Brands A, C, D and E, while sequences assigned to *Enterococcus* were detected in Brands B and D. DNA sequences assigned to *L. monocytogenes* were not detected in any samples. The coliform category was created by summing Bracken abundance estimates for bacterial genera identified by Health Canada [58] as selected coliform bacteria (*Budvicia*, *Citrobacter*, *Enterobacter*, *Erwinia*, *Klebsiella*, *Leclercia*, *Pantoea* and *Serratia*). *Escherichia* was excluded from the coliform group to avoid double counting.

**Table 3.** Relative abundance (%) of classified sequencing reads assigned to *E. coli*, coliforms, *Enterococcus* spp., *Salmonella* and *P. aeruginosa* within the enriched bacterial communities of five packaged ice samples. Values represent the percentage of reads assigned to each micro-organism relative to the total classified reads identified using Bracken. Brands A, B, C and D are non-IPIA accredited; Brand E is IPIA accredited. *Listeria* spp. was not detected

		Percentage of total reads				
Packaged ice	Brand	*E. coli*	Coliform^*^	*Enterococcus*	*Salmonella*	*P. aeruginosa*
Non-IPIA accredited	A	0.005	0.017	0	0.005	0.026
	B	0.002	0.058	0.001	0	0.057
	C	0.011	0.073	0	0.013	0.378
	D	0.003	0.017	0.001	0.003	0.012
IPIA accredited	E	0.004	0.018	0	0.004	0.013

*Coliform-associated abundance represents the sum of reads assigned to selected coliform genera as defined by Health Canada (*Budvicia*,* Citrobacter*,* Enterobacter*,* Erwinia*,* Klebsiella*,* Leclercia*,* Pantoea* and *Serratia*).

In several brands, pathogen DNA was detected through sequencing despite the absence of viable cells recovered via membrane filtration. Several methodological differences likely contribute to the observed differences between the culture-based and metagenomic sequencing. Culture-based analyses were performed on individual 100 ml samples, whereas metagenomic sequencing was conducted on an enriched composite sample from 500 ml of melted ice. Consequently, differences between the two methods likely reflect the combined effects of increased sample volume, sample composition and enrichment. Furthermore, because DNA extraction was performed following enrichment, the sequencing results cannot distinguish between DNA originating from viable, VBNC or dead cells [59]. Nevertheless, the detection of DNA assigned to bacterial pathogens suggests that these organisms may have been present at some stage during ice production or handling, reinforcing the importance of effective sanitation and contamination control throughout the production process. Currently, IPIA regulations (PIQCS) do not incorporate genome sequencing as part of compliance or enforcement [11]. Consequently, these findings suggest that reliance solely on culture-based filtration methods using 100 ml sample volumes may underestimate pathogen presence in packaged ice, particularly when micro-organisms are present at low abundance or in stressed or injured states.

Although *P. aeruginosa* was not recovered using traditional culture-based methods, its genomic detection is notable given its known ability to persist in water systems through biofilm formation and survival under low-nutrient or disinfected conditions [60, 61]. The presence of *Pseudomonas* spp. may therefore serve as an indicator of biofilm development and microbial persistence within ice production environments, potentially facilitating the survival of other micro-organisms and reducing the effectiveness of sanitation measures.

### Metagenomic diversity

While targeted genomic analysis revealed the presence of specific foodborne and indicator organisms, broader metagenomic profiling was conducted to characterize overall microbial community composition across packaged ice brands. Fig. 1 displays the relative abundance of the enriched bacterial genera greater than or equal to 1% of the metagenome identified in each packaged ice brand. *Stenotrophomonas* was the dominant genus in three brands, accounting for 95.3% of total reads in Brand A, 74.4% in Brand D and 97.2% in Brand E. Brand B was dominated by *Sphingobium* (56.1%). At the same time, *Ralstonia* comprised 93.9% of reads in Brand C. The ‘Other’ category includes genera with lower relative abundance not represented among the top five. The total number of genera identified was 111 in Brand A, 693 in Brand B, 415 in Brand C, 78 in Brand D and 91 in Brand E. Although the total number of genera detected was high in some brands, for example, 693 in Brand B, the enriched microbial community structure was often dominated by a single genus. While numerous other genera were identified, they were present at a relatively low abundance following enrichment. This dominance may reflect the preferential growth of certain bacteria under the enrichment conditions rather than their relative abundance in the original ice samples.

**Fig. 1.**
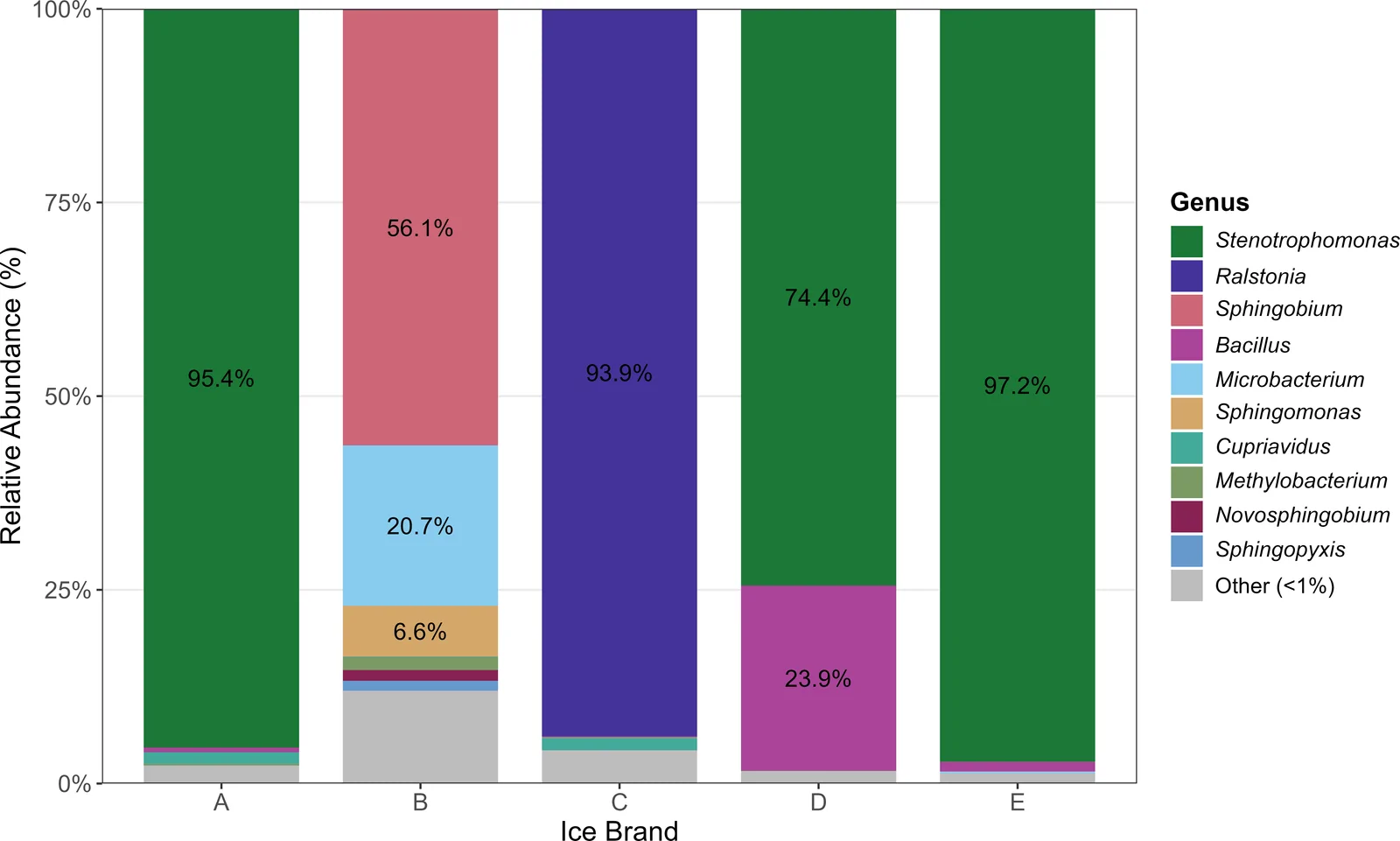
Relative abundance of bacterial genera (<1%) identified in the enriched microbial communities of five packaged ice brands (A–E). Colours represent bacterial genera, and values indicate their relative abundance (%). Brands A–D represent non-IPIA-accredited packaged ice brands, while Brand E represents the IPIA-accredited brand.

The dominant species following enrichment in Brands A, D and E was *Stenotrophomonas maltophilia*, a known environmental organism and an emerging multidrug-resistant opportunistic pathogen [62]. Although it has low inherent virulence, *S. maltophilia* has been detected in water systems, including hospital faucets, sinks and ice machines, being implicated in infections among immunocompromised individuals [63]. Its ability to persist on surfaces, particularly through biofilm formation, contributes to its resilience [62]. While it does not actively grow at refrigeration temperatures (4℃), it remains viable [64]. Notably, our metagenomic analysis detects DNA which may originate from both viable and non-viable cells. However, *S. maltophilia* consistent detection across multiple brands and relatively high abundance in three brands may indicate the persistence of biofilm-associated micro-organisms and highlight the importance of effective sanitation and monitoring practices in commercial ice production environments.

Sample C contained 93.9% of *Ralstonia*, with *Ralstonia pickettii* comprising 60% and *Ralstonia insidiosa* 12% of the genus. These species are commonly found in various water sources, including municipal, bottled and hospital water supplies, as well as in ultra-pure and high-purity water systems used in laboratories and industrial settings. Their adaptability to low-nutrient environments allows them to persist even in treated or filtered water [65]. *R. pickettii* is an aerobic, non-fermenting Gram-negative bacilli and an emerging opportunistic pathogen. Though generally low in virulence, it poses significant risks to immunocompromised individuals, where infections can result in mortality [65]. The high abundance of *Ralstonia* in sample C may reflect environmental contamination. Given its ability to persist in water systems and its relevance as an opportunistic micro-organism, this finding highlights the importance of maintaining effective sanitation and microbial monitoring practices in packaged ice production.

In Sample D, *Bacillus* accounted for 23.9% of the total microbial composition, with *Bacillus altitudinis* and *Bacillus cereus* as the dominant species. Members of the *Bacillus* genus are spore-forming bacteria that are commonly found in soil, air, water and processed foods. Their ability to form endospores makes them highly resilient in adverse environmental conditions, including cold storage and disinfection processes [66]. *B. cereus* is a well-known foodborne pathogen capable of producing enterotoxins and emetic toxins, leading to diarrheal and emetic food poisoning. Its spores can survive freezing, making it a concern in refrigerated and frozen food products, including ice [67, 68]. However, bacteria were extracted through enriching the filters, and their original abundance may have been low. Consequently, the overall risk posed by the organisms would remain low if proper food handling and sanitation practices were followed. However, as ice is commonly used in beverages and other food applications, there is potential for it to melt and reach temperatures that may support bacterial viability or growth. While this was not directly evaluated in the present study, such scenarios highlight the importance of assessing microbial risks throughout the food handling process.

*B. altitudinis*, while less studied, has also been isolated from water sources and is known for its biofilm-forming ability as well as AMR [69, 70]. DNA assigned to *Bacillus* spp., including *B. cereus*, was identified following enrichment and metagenomic sequencing. Although *B. cereus* is a recognized foodborne pathogen capable of producing heat-stable toxins and forming resistant spores, the sequencing approach used in this study does not confirm the presence of viable organisms, spore germination or toxin production. However, the detection of *Bacillus*-associated DNA following enrichment suggests that members of this genus may be present within the bacterial populations recovered from packaged ice and warrants further investigation using targeted culture-based or molecular approaches.

In Brand B, *Sphingobium* accounted for 56.1% of the total reads, with *Sphingobium yanoikuyae* as the dominant species. These Gram-negative, aerobic bacteria are commonly found in water and soil [71]. Although Brand B exhibited a high number of detected genera, only a small proportion of these are known to be of immediate public health concern. However, the presence of water-associated bacteria like *Sphingobium* may reflect gaps in filtration or sanitation practices. Given the limited information available on the potential health impacts of this genus in drinking or food-related applications, further research is needed to better characterize its persistence, pathogenic potential and relevance in ice and water systems.

### Antimicrobial resistance

AMRFinderPlus revealed a diverse repertoire of putative resistance determinants across all packaged ice brands (Fig. 2). Brand D harboured the highest number of putative resistance genes (14 genes), followed by the IPIA-accredited Brand E (13 genes), Brands A and C (5 genes), and Brand B had the lowest (1 gene). Genes predicted to confer *β*-lactam resistance, which are critically important antibiotics used as first-line treatments for many bacterial infections [72], were most frequently detected across Brands A–E. Overall, putative determinants for *β*-lactams and aminoglycosides were frequently identified resistance classes, together accounting for over half of all detected genes, with the following distribution: *β*-lactam (33.33%), aminoglycoside (17.95%), glycopeptide (10.26%), streptothricin (7.69%), tetracycline (7.69%), macrolide (7.69%), streptogramin (5.13%), fosfomycin (2.56%), lincosamide (2.56%) and pleuromutilin (2.56%). Brands D and E exhibited the greatest diversity of these putative sequences, sharing genetic markers associated with potential resistance to glycopeptides, a last-resort antibiotic, as well as tetracycline (first-line antibiotic) and streptothricin [73, 74]. In contrast, Brands B and C showed the lowest diversity, each carrying only putative determinants for *β*-lactam resistance. Crucially, Brand E was the only sample containing genetic sequences associated with macrolide, lincosamide, streptogramin and pleuromutilin resistance. Emphasizing their significance to human medical interventions, the WHO categorizes these antimicrobial classes as critically important (macrolides), highly important (lincosamides and streptogramins) and important (pleuromutilin) [75].

**Fig. 2.**
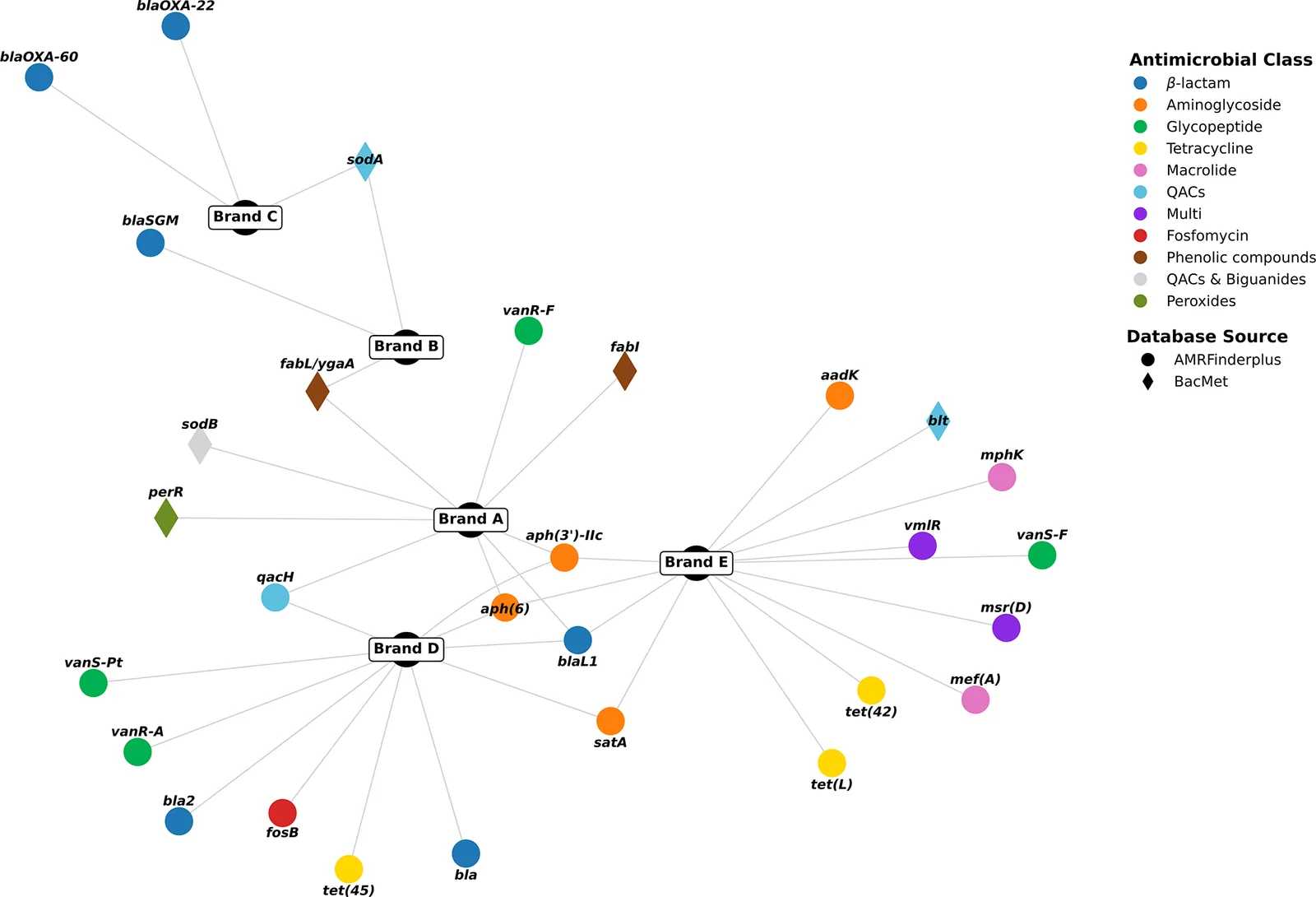
Network analysis of AMR genes across ice brands. Large nodes represent sampled brands (Brand E is IPIA-accredited; Brands A, B, C and D are non-accredited). Small nodes represent the identified resistance genes, colour-coded by their respective biocide or antibiotic class. Node shapes denote the reference database: circles indicate genes identified via AMRFinderPlus, while diamonds represent biocide resistance genes identified via BacMet. The ‘Multi’ class indicates genes conferring resistance to more than one class of antibiotics. Connection lines represent the detection frequency of each gene; solid lines represent a single detection (*n*=1), and dashed lines indicate multiple detections (*n*>1).

Metagenomic analyses using BacMet identified a total of 8 putative genes (Fig. 2) predicted to confer resistance to biocides, including quaternary ammonium compounds (QACs), peroxides and phenolic compounds. Specifically, the non-accredited brands (A, B and C) contained various genes including *fabI*, *fabL/ygaA*, *sodB*, *perR* and *sodA*. Brand D carried the *qacH* gene, while the accredited Brand E harboured the *blt* gene, both of which are associated with QAC resistance [76].

Interestingly, Brand E is subject to IPIA accreditation, which is based on GMPs, HACCP protocol and bacteriological testing to ensure compliance with strict PIQCS microbial criteria [38]. However, our results showed that compliance with food safety standards does not necessarily result in reduced AMR gene prevalence. We hypothesize that the detection of putative resistance determinants in accredited products might reflect selective pressure exerted by routine sanitation and disinfection programmes, which could favour the persistence of environmental micro-organisms possessing intrinsic resistance and stress adaptation mechanisms [77]. However, specific data regarding sanitizer exposure, concentration, frequency and production facility practices were not collected; this potential relationship remains speculative and warrants further investigation. In addition, biofilm-associated communities within water and processing systems may contribute to the maintenance and exchange of genetic resistance determinants, even in environments with strong hygienic controls [78–80]. Although AMR determinants have previously been reported in drinking water sources [81, 82], this study provides novel baseline data regarding their occurrence in packaged ice. These findings raise the possibility that packaged ice could represent an overlooked route of AMR exposure. However, establishing its potential role in AMR dissemination would require evidence of bacterial viability, mobility and transferability of the detected resistance determinants and clearly defined exposure pathways. Future risk assessment frameworks should also consider the broader microbial ecology of packaged ice, including the potential contribution of biofilm-mediated persistence to the maintenance and dissemination of AMR [83].

### Identification, bioinformatics and biofilm formation of *Pseudomonas* spp.

A total of 14 *Pseudomonas* strains were isolated from Brand E. All 14 isolates exhibited fluorescence under UV light, consistent with characteristics of *Pseudomonas fluorescens*. DNA extraction and sequencing, followed by taxonomic identification using the BVBRC, confirmed the species as *P. lactis*. This bacterium, first discovered by von Neubeck *et al*. [84] following its isolation from raw bovine milk, belongs to the *P. fluorescens* group, is psychrotrophic and grows at 4 °C. The capacity of *P. lactis* to persist and grow at refrigeration temperatures is particularly relevant in the context of ice production and storage, which typically occurs at or near 4 °C. Given the limited characterization of this species, the *P. lactis* isolates were subjected to further phenotypic and genotypic analysis. Furthermore, pairwise ANI analysis (Fig. 3) revealed that all *P. lactis* isolates shared high genomic similarity, with ANI values ranging from 99.93 to 100%. According to established taxonomic thresholds, an ANI of ≥95% indicates that the isolates are highly related at the genomic level and likely belong to the same species [40]. The near-identical genomic content among isolates suggests limited nt divergence, reflecting a clonal structure. To further assess genomic relatedness, pangenome analysis with Roary identified a total of 6,727 genes across all 14 isolates. Of these, 6,230 genes (92.6%) were present in all genomes. The remaining 497 genes represented only minor genomic variation among the isolates. Consistent with these findings, SNP distance analysis (Table S2, available in the online Supplementary Material) revealed low pairwise genetic distances ranging from 33 to 112 SNPs. These distances fall within previously reported thresholds used to define clonality realted isolated, which range from 15 up to 200 SNPs [85]. This high genomic similarity aligns with the repeated recovery of *P. lactis* from the same ice brand over several months, suggesting a persistent contamination source rather than repeated independent introductions. Together, the ANI and pangenome analyses suggest that a single, relatively stable lineage of *P. lactis* was able to persist within the ice production environment.

**Fig. 3.**
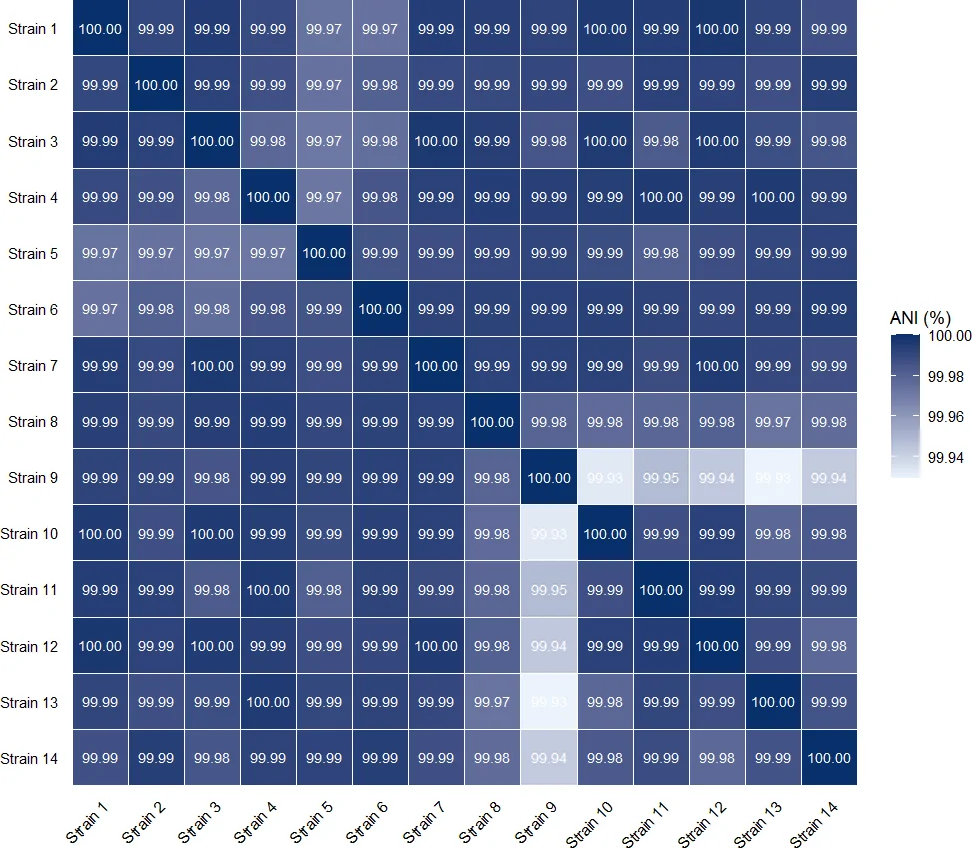
ANI heat map showing pairwise genomic similarity among 14 P. lactis isolates recovered from packaged ice. Each cell represents the ANI value (%) between two genomes, where darker blue shading indicates higher nt identity and lighter blue indicates lower identity. Values along the diagonal represent self-comparisons (100% identity). The colour scale legend corresponds to the range of ANI values observed.

The abriTAMR analysis indicated that no AMR genes were detected under the parameters applied. In contrast, analysis using the BacMet database, which identifies biocide genes associated with resistance to biocides and heavy metals, revealed a total of 24 putative resistance determinants present across the isolates. All *P. lactis* carried the genes *recG*, *cueA*, *copS*, *copR*, *copD*, *copC* and *chrA*, representing the metal resistance class. The QAC resistance class was represented by the bcrC gene. Determinants classified as multiclass included *ttgB*, *ruvB*, *oscA*, *meF*, *mexE*, *mepB*, *emhR*, *emhC*, *emhB* and *acrJ/yjcG*. Finally, the phenolic compound resistance class included *mexT* and *mexK*. All isolates carried three copies of *copC and* two copies of *recG* and *ruvB*.

Genes classified as multiclass are of particular concern as they are putatively associated with tolerance or resistance to compounds from multiple biocide classes. Many of these mechanisms rely on efflux pumps, which can expel antimicrobial compounds from the cell [86]. Among these, the MexEF-OprN efflux system is a well-described multidrug pump found in several Gram-negative species, as well as the opportunistic pathogen *P. aeruginosa* [87]. The MexEF-OprN is associated with resistance to fluoroquinolones, chloramphenicol and trimethoprim. The regulation of the MexEF-OprN operon is multifaceted, with the activator *mexT* serving as a regulator [88], which was present in all *P. lactis* isolates. Additionally, the isolates encoded *mexK*, which forms part of the MexJK operon, another multidrug efflux pump system [89].

Metal resistance is particularly relevant in water systems because copper is widely used in water and food processing facility equipment. As a result, micro-organisms that possess copper resistance mechanisms may gain a selective advantage in these environments. Multiple copper-associated resistance determinants were identified in the *P. lactis* isolates. The presence of the *copABCD* operon and its associated regulatory genes (*copR/copS*) provides the genomic potential for copper sequestration and export, which could theoretically aid in surviving exposure to copper stressors commonly encountered in water distribution and food processing systems [90, 91]. However, it is important to note that sequence annotation alone does not confirm functional resistance. These findings represent hypothesized capabilities, and phenotypic assays are required to confirm the protective role. Furthermore, other metal-associated genes detected include *chrA*, which is frequently annotated for chromate resistance [91], and *recG*, a DNA repair helicase [92]. While *recG* can mitigate DNA damage induced by toxic oxyanions such as chromium, tellurium and selenium, it fundamentally performs essential physiological and housekeeping functions related to general DNA replication and repair [93, 94].

We hypothesized that persistent contamination of *P. lactis* in the ice environment may arise from biofilm formation. Temperature is a well-recognized regulator of biofilm development, and bacterial species can exhibit reduced or inhibited biofilm formation under cold conditions [13, 95]. To investigate the effect of cold temperature on our isolates, the biofilm-forming ability of the 14 *P*. *lactis* strains was determined at 4 and 10 °C (Fig. 4). Although the *P. lactis* isolates were highly clonal based on ANI analysis, their biofilm formation capacity varied notably. A one-way ANOVA revealed a significant effect of temperature on biofilm production (*P*=1.47×10⁻⁵), with isolates producing significantly greater biofilm biomass at 4 °C (mean OD=0.283) than at 10 °C (mean OD=0.198). These results indicate that colder conditions promoted stronger biofilm formation in these isolates. Strain ID 4 was the only isolate that did not produce a strong biofilm at 4 °C. Comparative genomics using Roary identified 71 gene differences between strain 4 (weakest former) and strain 8 (strongest former). Notably, the weakest former, strain 4, showed no differences in biofilm genes compared to the stronger formers. In addition, the pan-genome comparison revealed 496 accessory genes present in fewer than 14 isolates, representing ~0.07% of the genomic difference between strains. According to the BBSdb annotation, all 14 isolates encoded 119 known biofilm-associated genes, except for strains 3 and 7 lacking *argG* and strain 11 lacking *eutB*. However, because the weakest biofilm former possessed all 119 genes, these specific genomic absences do not explain the observed strain-specific variation in biofilm formation at different temperatures. Hence, additional functional and gene expression analyses are required.

**Fig. 4.**
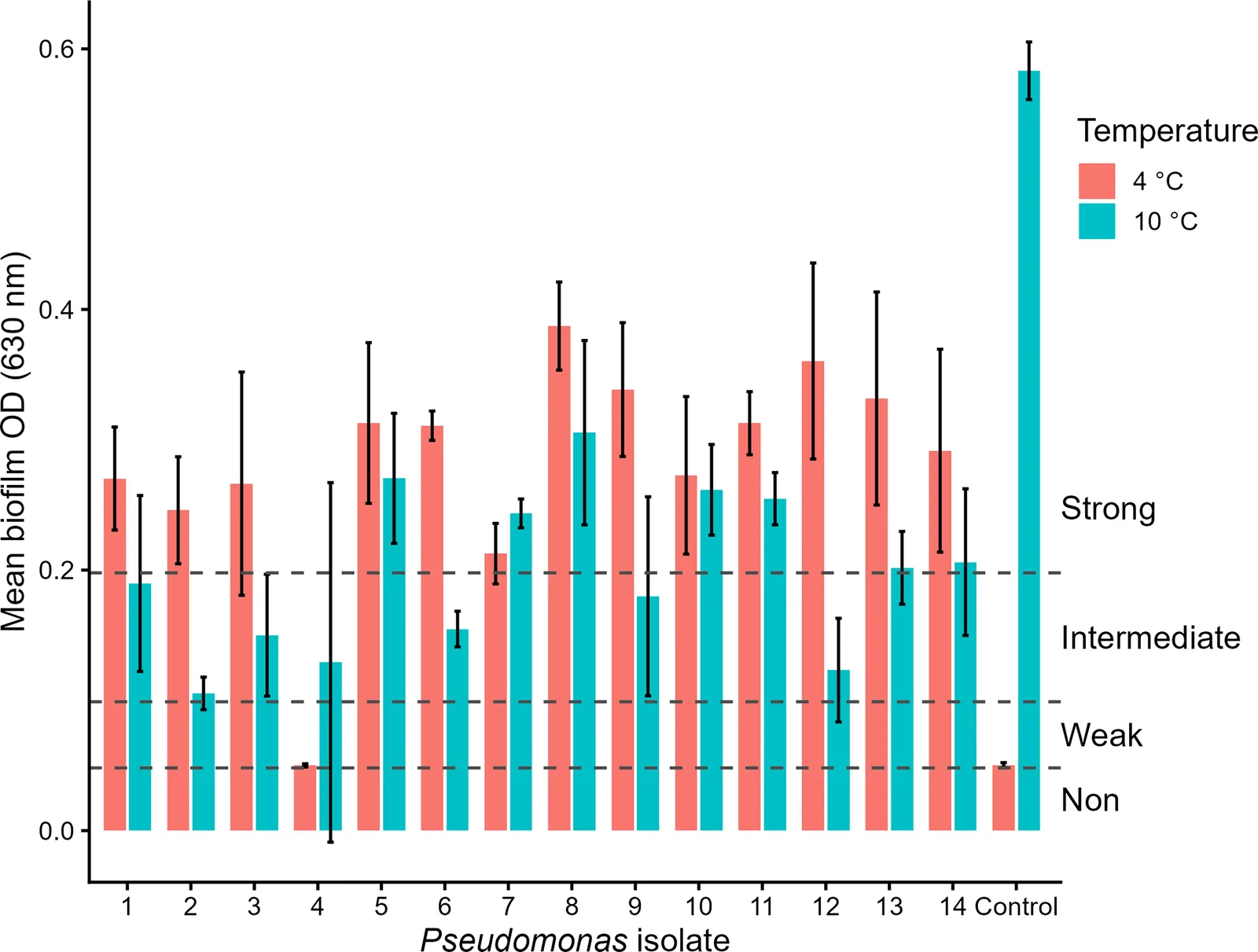
*Pseudomonas* isolates biofilm formation at 4 and 10 °C after 8 days. Mean biofilm biomass (OD₆₃₀) measured at 4 °C (pink bars) and 10 °C (teal bars), with error bars representing sd from three biological replicates for each isolate. Horizontal lines were statistically determined using the negative controls from each temperature and represent division between non-biofilm formers (bottom; OD<ODc), weak (lower middle; ODc<OD<2 ODc), intermediate (upper middle; 2 ODc<OD<4 ODc) and strong (top; 4 ODc<OD), with *Raoultella* used as a control. Within the temperatures 4 °C showed a significant effect in biofilm formation (*P*=1.47×10⁻⁵).

Together, the phylogenetic analysis, cold-temperature biofilm formation assay and the presence of multiple metal- and multidrug-resistance genes suggest that the *P. lactis* isolated from ice represent a resilient, environmentally adapted lineage capable of persisting in ice-processing systems. Although genetically similar, the isolates differed in their ability to form biofilms at 4 and 10 °C, suggesting that strain-level variation may contribute to long-term survival in cold, nutrient-limited environments. The identification of biocide and metal resistance genes further supports the potential for these strains to survive cleaning practices and other stresses typical of ice equipment.

Overall, these findings indicate that persistent contamination may be driven by bacterial traits not routinely monitored in the industry, including biofilm-associated protection and microbial interactions that can shelter or inhibit foodborne pathogens. This highlights the need to incorporate environmental sampling that accounts for biofilm formation, alongside products specifically designated to target and destroy biofilms. For instance, Koti *et al*. [36] recommend updating industry sanitation SOPs to include a mandatory multi-tiered approach. They also suggest integrating physical mechanical scrubbing to break the biofilm matrix prior to chemical application, prioritizing specialized biofilm-disrupting sanitizers such as peracetic acid (BioDestroy®) and establishing stricter hygiene assessment thresholds rather than relying on visual inspection alone.

### Conclusions

Of the five packaged ice brands evaluated, one with IPIA accreditation and four without, the IPIA-accredited brand, Brand E, met all microbial standards outlined by PIQCS. Non-compliant samples were identified among Brands A, C and D. However, the frequency of non-compliance varied among brands, with Brand C exhibiting the highest proportion of non-compliant samples (55%), compared with 6.5 and 5% for Brands A and D, respectively. Although IPIA standards are voluntary, they currently provide important guidance for ice producers seeking to ensure product safety. Overall, most individual samples met conventional microbial quality indicators; however, metagenomic and genomic analyses provided additional insight into the broader microbial communities, AMR determinants and persistence-associated traits present in commercially available ice.

Beyond conventional microbial quality indicators, metagenomic characterization and biofilm assessment of *P. lactis* provided further insight into microbial persistence and the presence of AMR determinants in packaged ice. The repeated isolation of highly related *P. lactis* carrying genes associated with biocide and metal resistance, together with their ability to form biofilms at refrigeration temperatures, suggests traits that may facilitate persistence in ice processing environments. The characterization of *Pseudomonas* spp. provided valuable information for microbial risk assessment and environmental monitoring in ice production environments. Furthermore, the widespread detection of putative genes associated with antimicrobial and biocide resistance across all sampled brands suggests packaged ice may represent a potential reservoir of resistance associated with genetic determinants. Biofilm formation may contribute to microbial persistence by sheltering micro-organisms from environmental stresses and reducing the effectiveness of sanitation practices. The implementation of targeted anti-biofilm interventions during sanitation procedures may help reduce bacterial persistence and limit the exchange of resistance genes within microbial communities. These findings should be interpreted with the limitations of the study. Metagenomic sequencing was performed on a single enriched composite sample from each brand. Enrichment prior to DNA extraction may have altered the relative composition of the microbial community by preferentially promoting the growth of certain micro-organisms. While the detection of microbial DNA does not confirm the presence of viable cells, these findings provide valuable baseline data for future surveillance, risk assessment and a better understanding of the microbial ecology of packaged ice. Collectively, these findings support the consideration of more standardized and enforceable microbiological requirements for packaged ice in Canada.

## Supplementary material

10.1099/mic.0.001780 Table S1.

## Abbreviations

AMR: antimicrobial resistance
ANI: average nt identity
BBSdb: Bacteria Biofilm Sequence Database
BPB: Butterfield’s Phosphate Buffer
BPW: buffered peptone water
CFIA: Canadian Food Inspection Agency
CV: crystal violet
FDA: Food and Drug Administration
GMPs: Good Manufacturing Practices
HACCP: Hazard Analysis and Critical Control Point
IPIA: International Packaged Ice Association
LBNS: Luria–Bertani nutrient broth without salt
NCBI: National Center for Biotechnology Information
ODc: OD cut-off
PIQCS: Packaged Ice Quality Control Standards
QAC: quaternary ammonium compound
RV: Rappaport-Vassiliadis
SOPs: Standard Operating Procedures
TT: tetrathionate
VBNC: viable but non-culturable
WHO: World Health Organization
